# Phenylpyrazalopyrimidines as Tyrosine Kinase Inhibitors: Synthesis, Antiproliferative Activity, and Molecular Simulations

**DOI:** 10.3390/molecules25092135

**Published:** 2020-05-02

**Authors:** Bhupender S. Chhikara, Sajda Ashraf, Saghar Mozaffari, Nicole St. Jeans, Dindyal Mandal, Rakesh Kumar Tiwari, Zaheer Ul-Haq, Keykavous Parang

**Affiliations:** 1 Department of Chemistry, Aditi Mahavidyalaya, University of Delhi, Bawana, Delhi 110039, India; bschhikara@gmail.com; 2 Department of Biomedical and Pharmaceutical Sciences, College of Pharmacy, University of Rhode Island, Rhode Island, Kingston, RI 02881, USA; nicolestjean@ymail.com (N.S.J.); mandal@chapman.edu (D.M.); tiwari@chapman.edu (R.K.T.); 3 Dr. Panjwani Center for Molecular Medicine and Drug Research, ICCBS, University of Karachi, Karachi 75210, Pakistan; sajda.ashraf@yahoo.com; 4 Center For Targeted Drug Delivery, Department of Biomedical and Pharmaceutical Sciences, Chapman University School of Pharmacy, Harry and Diane Rinker Health Science Campus, California, Irvine, CA 92618, USA; mozaf100@mail.chapman.edu

**Keywords:** protein kinase, phenylpyrazolopyrimidine, antiproliferative activity, enzyme inhibition, molecular simulation

## Abstract

*N*1-(α,β-Alkene)-substituted phenylpyrazolopyrimidine derivatives with acetyl and functionalized phenyl groups at α- and β-positions, respectively, were synthesized by the reaction of 3-phenylpyrazolopyrimidine (PhPP) with bromoacetone, followed by a chalcone reaction with differently substituted aromatic aldehydes. The Src kinase enzyme assay revealed modest inhibitory activity (half maximal inhibitory concentration, IC_50_ = 21.7–192.1 µM) by a number of PhPP derivatives. Antiproliferative activity of the compounds was evaluated on human leukemia (CCRF-CEM), human ovarian adenocarcinoma (SK-OV-3), breast carcinoma (MDA-MB-231), and colon adenocarcinoma (HT-29) cells in vitro. 4-Chlorophenyl carbo-enyl substituted 3-phenylpyrazolopyrimidine (**10**) inhibited the cell proliferation of HT-29 and SK-OV-3 by 90% and 79%, respectively, at a concentration of 50 µM after 96 h incubation. The compound showed modest inhibitory activity against c-Src (IC_50_ = 60.4 µM), Btk (IC_50_ = 90.5 µM), and Lck (IC_50_ = 110 µM), while it showed no activity against Abl1, Akt1, Alk, Braf, Cdk2, and PKCa. In combination with target selection and kinase profiling assay, extensive theoretical studies were carried out to explore the selectivity behavior of compound **10**. Specific interactions were also explored by examining the changing trends of interactions of tyrosine kinases with the phenylpyrazolopyrimidine derivative. The results showed good agreement with the experimental selectivity pattern among c-Src, Btk, and Lck.

## 1. Introduction

Cancer cells show the overexpression of different protein tyrosine kinases (PTKs). These enzymes catalyze the phosphorylation of many protein substrates by the transfer of the γ-phosphate group from ATP to specific tyrosine residues. PTKs have critical roles in signal transduction pathways.

Src family kinases (SFKs) have nine different PTKs, including c-Src, c-Yes, Fyn, Lck, Lyn, Hck, Frk, Blk, and c-Fgr of which Src is the prototype [1]. SFKs have essential roles in the regulation of a wide variety of normal cellular signal transduction pathways, such as cell division, differentiation, growth factor signaling, survival, adhesion, migration, and invasion [2]. Src tyrosine kinase expression is frequently elevated in several tumors, including breast, colon, prostate, lung, ovary, and pancreas, when it is compared with the adjacent healthy tissues. Src kinase was shown to be the critical modulator of cancer cell metastasis and invasion [3].

**Figure 1:**
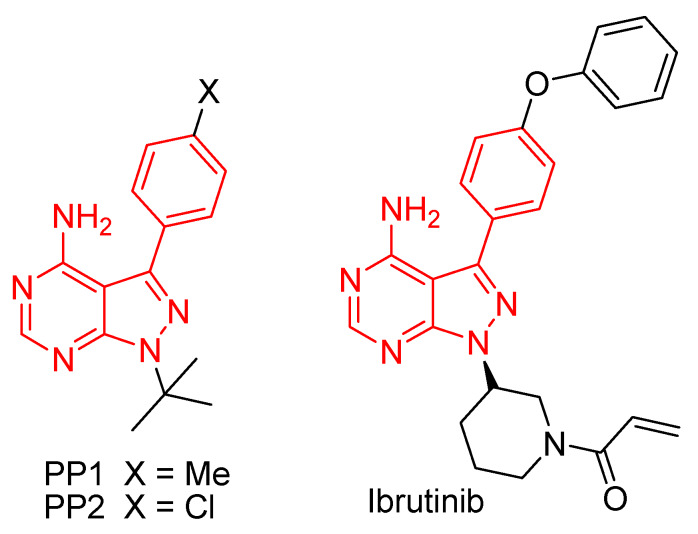
Chemical structures of 3-phenylpyrazolopyrimidine (PhPP) derivatives (PP1 and PP2) and Ibrutinib.

In recent years, a number of small cell-permeable inhibitors of protein kinases were investigated for the treatment of different cancers [4]. Different small molecules that were actively investigated as kinase inhibitor include quinazolines [5], quinolinecarbonitriles [6], pyrazolopyrimidines [7,8], imidazo[1,5-a]pyrazines [9], Calixarene [10], benzotriazines [11,12], indoles [13], and ATP-phosphopeptide conjugates [14]. The research effort led to the generation of small molecules for the treatment of different malignancies. Imatinib, a 2-phenylaminopyrimidine, was approved for the treatment of chronic myelogenous leukemia (CML) and gastrointestinal stromal tumors (GISTs) [15]. Dasatinib (BMS-354825), a pyrimidinylthiazole derivative, was approved to use in patients with CML after imatinib treatment [16], and bosutinib (SKI-606), a 3-quinolinecarbonitrile analogue, is known to suppress migration and invasion of human breast cancer cells [17]. Ibrutinib (Figure 1) is a pyrazolopyrimidine-based [18] small-molecule kinase inhibitor that was approved for the treatment of B-cell cancers like mantle cell lymphoma, chronic lymphocytic leukemia, and Waldenström’s macroglobulinemia [19,20].

A number of crystallographic studies showed that adenosine 5′-(β,γ-imido)triphosphate (AMP-PNP), an ATP mimic, binds to the ATP-binding site of c-Src (Protein Data Bank (PDB) 2SRC) [21]. Furthermore, complexes of 3-phenylpyrazolopyrimidine (PhPP) derivatives (PP1 and PP2) (Figure 1) act as ATP-binding site inhibitors of Hck (PDB 1QCF) and Lck (PDB 1QPE) [22]. Indeed, the pyrazolopyrimidine core of PP1 and PP2 (Figure 1) mimics the adenine base of ATP in binding to the nucleotide-binding site. In another study [8], we showed that the 3-phenyl group interacts with a large hydrophobic pocket in the ATP-binding site, contributing significantly to Src kinase inhibitory activity. There is also another cavity formed from side chains of helix αC and helix αD that is normally occupied by a carbohydrate moiety followed by a triphosphate group of ATP. This cavity is mostly unfilled by the tert-butyl of 3-phenylpyrazolopyrimidines (PP1 and PP2). Furthermore, we investigated the variation of *N*1 substitution in 3-phenylpyrazolopyrimidines with different 1,2,3-triazoles containing hydrophobic residues to interact with amino acids of this cavity and contribute to the enhancement of Src kinase inhibitory potency [8]. The cysteine residue present in the cavity could be a potential target to bind with PhPP molecules irreversibly, leading to a potential increase in kinase inhibition.

**Figure 2:**
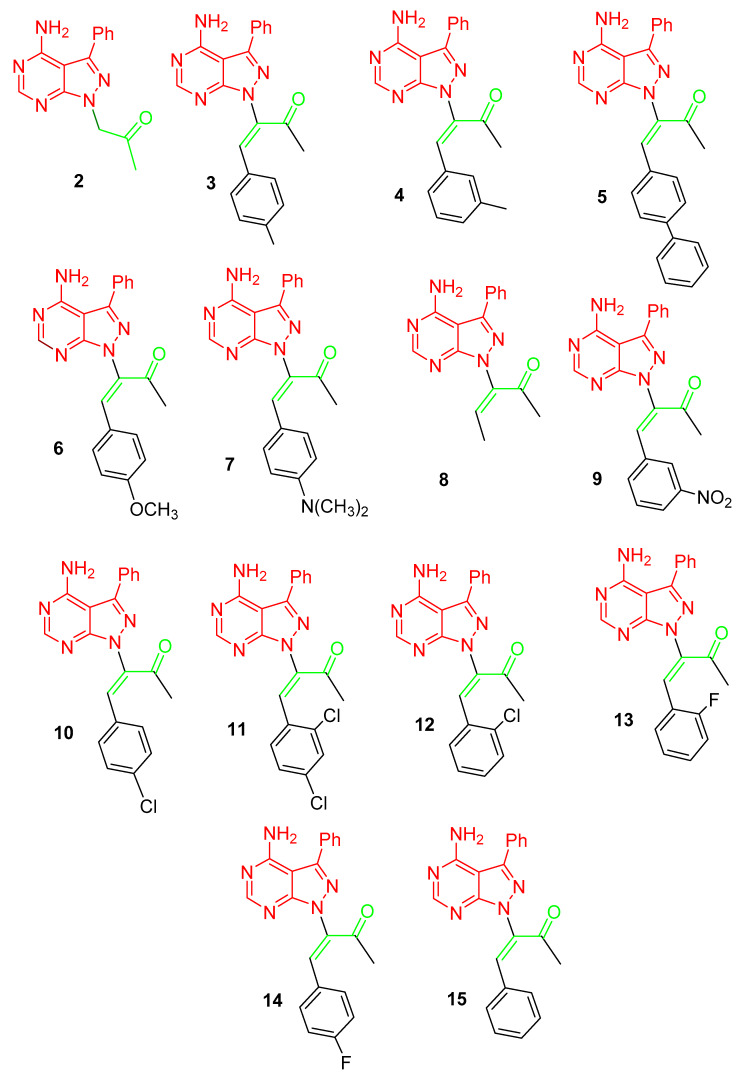
Chemical structures of *N*1-substituted PhPP with α-acetyl-β-phenyl-alkene groups (*E* and *Z* isomers).

Here, we describe the synthesis and evaluation of kinase inhibitory activity of *N*1-substituted PhPP with α-acetyl-β-phenyl-alkene groups (Figure 2). We hypothesized that this substitution at the *N*1 position of PhPP could contribute significantly to the kinase inhibition through generating specific interactions. We further extended our study to investigate the selectivity profile of one of the active compounds (**10**) with a panel of nine different kinases by applying MD and MMGBSA calculations. This study helped to reveal the inhibitory mechanism of PhPP derivatives based on their distinct structural characteristics required for its interaction with a specific kinase.

## 2. Results and Discussion

### 2.1. Chemical Synthesis

**Scheme 1:**
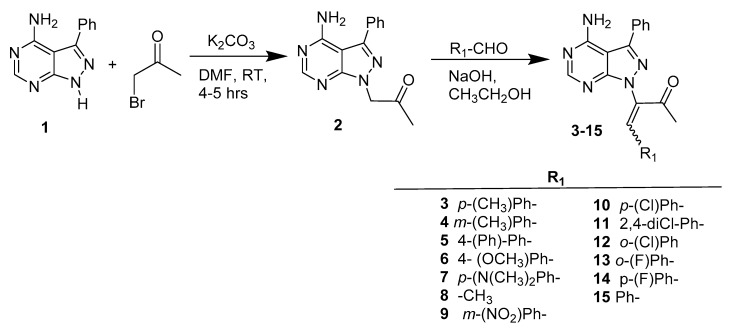
Chemical synthesis of PhPP derivatives.

The products were synthesized by following the chemical pathway shown in Scheme 1. The PhPP (**1**) was synthesized according to a reported procedure [23]. The N1 of **1** was substituted with bromoacetone in the presence of potassium carbonate as a base. Product **2** was confirmed by NMR, which showed the disappearance of the proton peak of NH at 8.07 ppm of **1** and appearance of the peak at 5.24 ppm and 2.21 ppm for CH_2_ and CH_3_, respectively, in ^1^H NMR. Furthermore, ^13^C NMR showed peaks at 201.34 ppm, 56.15 ppm, and 27.14 ppm representing the carbonyl group, CH_2_, and CH_3_, respectively, in product **2**.

Next, the double bond group was introduced by the chalcone reaction. The anion of **2** generated by sodium hydroxide was reacted with different benzaldehydes at room temperature to give product **3**. The ratio of starting material and amount of solvent defined the formation of product and side products. A higher concentration (i.e., less amount of solvent ethanol) and the presence of higher equivalents of **2** than the aldehyde lead to a second internal Michael reaction where the anion of **2** reacts with product **3**, leading to the formation of side product as indicated by Electrospray Ionization Mass Spectrometry (ESI-MS) at 659 Da (data not shown). In an optimum condition, the aldehyde and reactant **2** should be present in more than 1.4:1 equivalent, and solvent ethanol should be present in approximately 20 mL for 26 mg (0.1 mmol) of **2**. Out of two possible products after the conjugation of **2** with 4-methylbenzaldehyde, only product **3** was observed, suggesting the reaction of the carbanion of methylene (CH_2_) group between the carbonyl and nitrogen rather than that of methyl (CH_3_). The formation of product **3** was confirmed by ESI-MS by the presence of a mass peak at 370 Da [M + H]^+^. This was confirmed by ^1^H NMR, which showed the absence of a peak at 5.24 ppm for CH*_2_* of **2,** while the three protons for CH*_3_* were present at 2.31 ppm. Correspondingly, in ^13^C NMR, the peak at 56.15 ppm (assigned to *C*H_2_) was absent in **3** while the peak at 26.13 ppm (assigned to *C*H_3_ with slight upfield shift) was present. Furthermore, out of EZ possible isomers for the double bond, both the isomers were present as indicated by NOE NMR spectra, possibly by rotation introduced due to keto-enol tautomerism.

In the case of product **8**, the chalcone reaction was carried with precaution to avoid the formation of side products due to the presence of α-hydrogen in acetaldehyde. The anion of **2** was generated first with 1.1 equivalent of sodium hydroxide over extended time and added to the solution of acetaldehyde (1.2 equivalents). The reaction was quenched with acetic acid after 30 min. The product was confirmed by ESI-MS and NMR. The products were purified by HPLC and showed a purity of 95–99%.

### 2.2. Src-Kinase Assay

In vitro Src kinase assay for some of the compounds was conducted, as presented in Table 1. As results indicated, the compounds were moderately active against the Src kinase. Among all the tested compounds, compounds **6** and **4** were found to be the most potent with IC_50_ values of 21.7 and 24.7 µM, respectively. Compounds **3** and **7** exhibited IC_50_ values of 32.9 µM and 36.8, respectively. Compound **5** showed the lowest activity with an IC_50_ of 192.1 µM. Since the compounds showed only moderate Src kinase inhibitory activity, they were evaluated for antiproliferative activity first to find the lead compound for further kinase inhibitory assays.

### 2.3. Antiproliferative Activities

**Figure 3:**
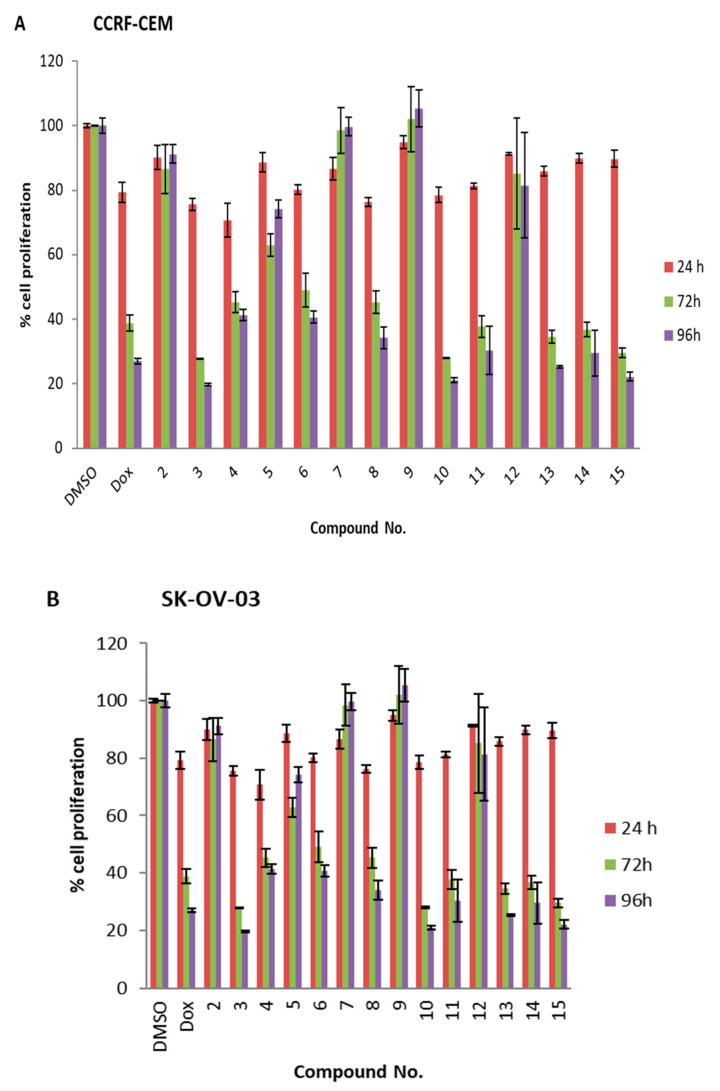

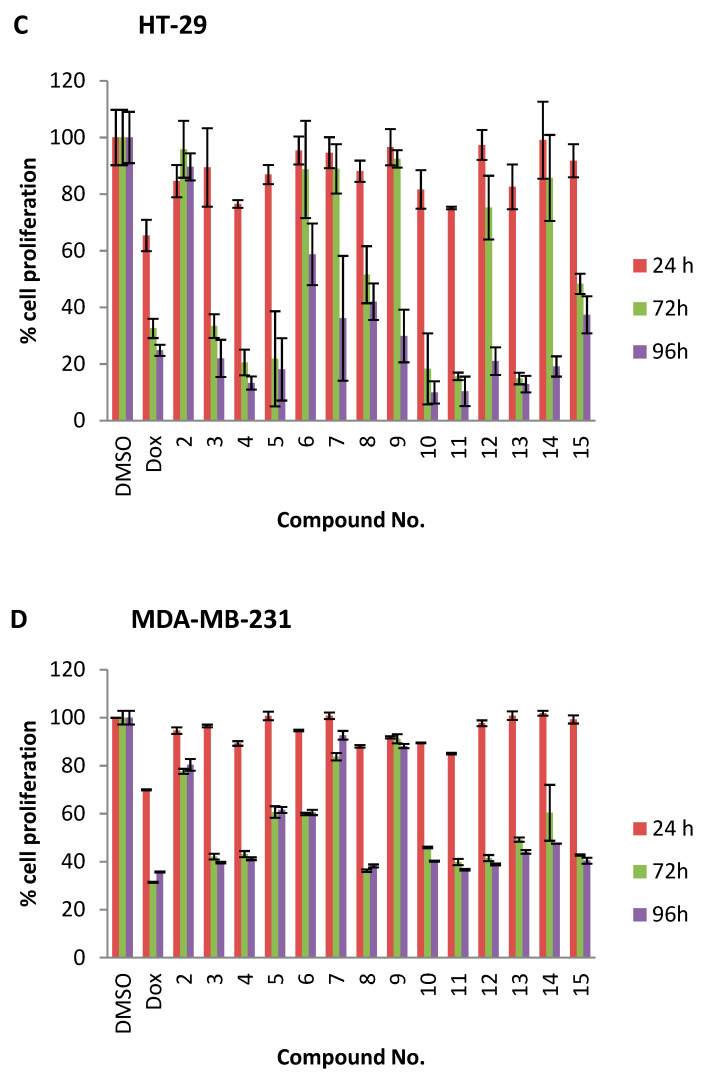
Antiproliferative activity of PhPP derivatives (50 µM) and doxorubicin (Dox) (1 µM) against CCRF-CEM (**A**), SK-OV-3 (**B**), HT-29 (**C**), and MDA-MB-231 (**D**) cell lines. All experiments were repeated in triplicate.

**Figure 4:**
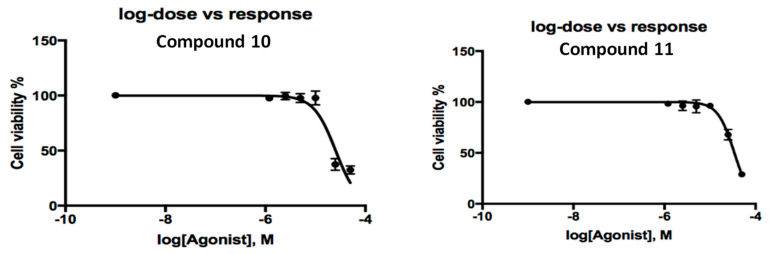
Dose-response curves of **10** (**left**) and **11** (**right**) after 72 h incubation against SK-OV-3 cells.

The effect of compounds on the cell proliferation of cancer cells was evaluated in vitro in a human leukemia cell line (CCRF-CEM) according to the previously reported procedures [24,25], breast adenocarcinoma (MDA-MB-231), ovarian adenocarcinoma (SK-OV-3), and colon adenocarcinoma (HT-29) cell lines up to 72 h. Doxorubicin (Dox) was used as a positive control as shown previously [26,27,28,29]. These cell lines were selected to determine the antiproliferative activity against diverse cancer cells. Compounds **10** and **11** consistently inhibited the cell proliferation 72–96 h in all cell lines. The percentage of cell proliferation was significantly reduced in HT-29 cells in the presence of **10** and **11** when compared with Dox and other compounds. Compound **10** was more effective in the order of HT-29 (90%) ˃ CCRF-CEM, SK-OV-3 (79%) ˃ MDA-MB-231 (60%), while Dox showed HT-29 (75%) ˃ CCRF-CEM, SK-OV-3 (73%) ˃ MDM-MB-231 (64%) after 96 h. Compounds **2**, **7**, **9**, and **12** were found to be inactive in reducing the cell proliferation in all the cell lines after 24–96 h while other compounds **3** and **13**–**15** exhibited comparable or slightly less antiproliferative activity (Figure 3A–D) than compounds **10** and **11** after 96 h. These data indicate that the presence of the chlorine group at the para position in compounds **10** and **11** were effective in improving the antiproliferative activities. However, the absence of phenyl and/or double bonds in compounds **2** and **8** significantly reduced the antiproliferative activities versus **10** and **11**. The presence of *p*-dimethylamino, *m*-nitro, and *o*-chlorine in **7**, **9**, and **12** were not productive while *o*-fluorine (**13**), *p*-fluorine (**14**), unsubstituted phenyl (**15**), and *p*-Cl (**10** and **11**) enhanced the antiproliferative activity. Compounds **5** and **6** with *p*-ph and *p*-methoxy exhibited antiproliferative activity against some of the cell lines, with **5** being more active in HT-29 and **6** in CCRF-CEM and SK-OV3 cells. However, their activity was generally less than compounds **3**, **10**, **11**, **13**, and **14**. Compounds **10** and **11** exhibited IC_50_ values of 25.5 µM and 34.3 µM, respectively, after 72 h incubation against SK-OV-3 cells (Figure 4).

Compound **10** was selected for further evaluation against a panel of kinases to determine the mechanism of activity. The results are shown in Table 2 against Abl1, Akt1, Alk, Braf, Btk, Cdk2, cSrc, Lck, and PKCa enzymes. The data indicate modest activity against Btk, c-Src, and Lck while the compound was inactive or showed very low inhibition activity against other kinases.

### 2.4. Molecular Modeling

To rationalize the activity of compound **10**, KEGG pathway and molecular dynamics simulation studies were utilized to explore the specific behavior of the *Z* configuration compound **10** against multiple kinases.

#### 2.4.1. Target Identification

**Figure 5:**
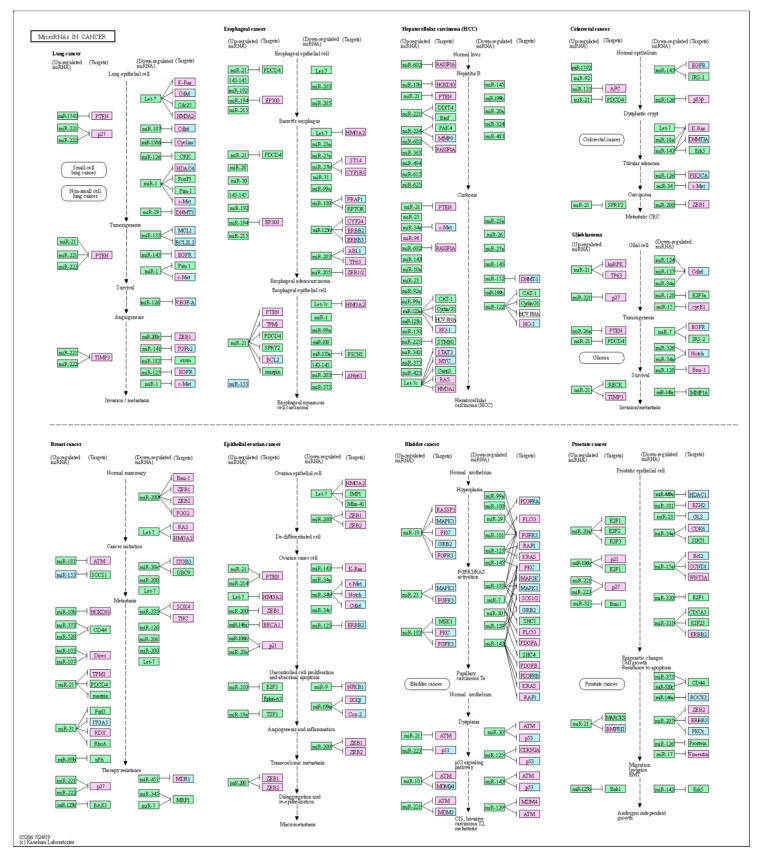
The KEGG pathway for ovarian cancer.

**Figure 6:**
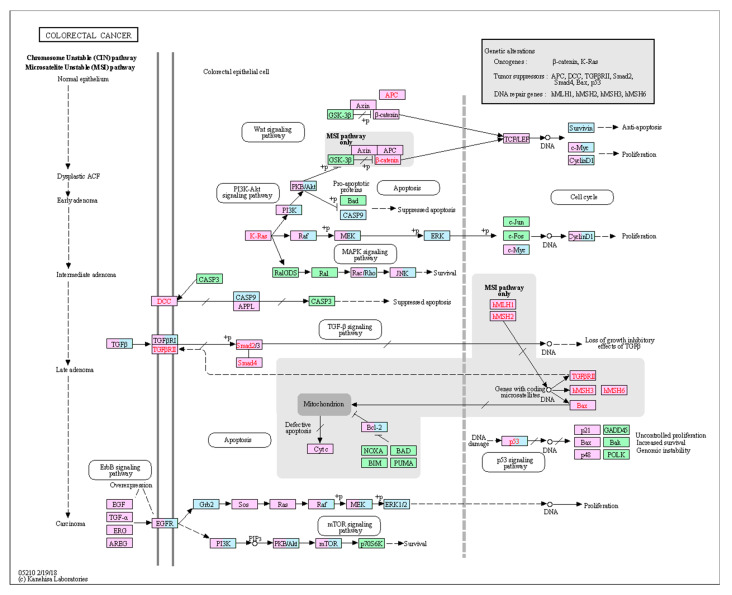
The KEGG pathway for colorectal cancer.

**Figure 7:**
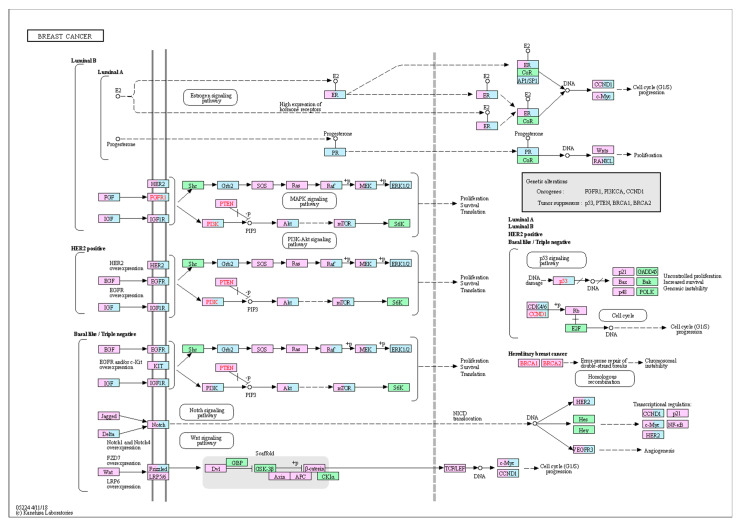
The KEGG pathway for breast cancer.

Conventional identification of drug targets is an expensive, time-consuming, and difficult process; only a few drug targets can be identified. In contrast, the computational method permits a great deal of analysis within a short period and brings a large number of potential drug targets from a pool of information [30]. In the present study, an integrated in silico approach was used to identify potential targets [31] for the active compound **10**. Initially, the disease search tool in the KEGG database was used against breast, ovarian, and colorectal cancer to extract the targets that may be involved in these diseases (Figure 5, Figure 6 and Figure 7) [32]. KEGG uses the knowledge of gene function and linking this information with advanced order functional information by using systematic analysis. The schematic presentation of the KEGG pathway shows genes marked as light-blue color as a drug target and genes marked as pink as associated with the disease, whereas when the gene is linked with both a disease and a drug target, its color is split into light blue and pink. There were several target proteins involved in one pathway; therefore, protein-drug association servers Similarity Ensemble Approach (SEA, http://sea.bkslab.org/) [33], Search Tool for the Retrieval of Interacting Genes (STRING, http://string-db.org) [34], and Search Tool for Interacting Chemicals (STITCH, http://stitch.embl.de/) [35] were used. The STRING database was used to explain the molecular function, biological processes, cellular components, and pathways of the target proteins. The SEA relates target proteins based on set-wise chemical similarity among their compounds. A total of 14 potential targets (Btk, Itk, c-Src, EGFR, Akt1, Fyn, Lyn, Lck, PKC, Abl1, Hck, Cdk2, Braf, and Her2) were selected based on the data obtained from these servers that further proved the reliability of text mining and molecular docking.

#### 2.4.2. Docking Studies

The known compounds that were already reported as inhibitors of the target proteins, as well as nature and crucial active site residues, were specified in their accessible complexes, used as a positive control. Prior to docking, validation of the software and docking conditions was performed by retrieving the control compounds from their crystal complexes and then redocking by MOE against their relevant targets. The redocking results are presented in Table 3. After validation, docking of compound **10** was performed with all 14 targets, and their docking scores were compared with the control in order to select a target with the highest docking score. We observed that compound **10** presented good scores against Btk, Itk, c-Src, EGFR, Akt1, Fyn, Lyn, Lck, PKC, and Abl1 kinase as compared to Hck, Cdk2, Braf, and Her2. The docking scores of compound **10** are presented in Table 4.

#### 2.4.3. Structure Architecture of Kinases

The structure of all protein kinase domains is composed of a small, typically β-stranded *N*-lobe linked with a small hinge region to a bigger α-helical *C*-lobe. In protein kinases, ATP binds to the cavity that lies between *N*- and C-terminal lobes creating the kinase activation site, where the adenine moiety of ATP is packed like a sandwich between hydrophobic residues, while it is associated with the hinge region by hydrophilic contact. Typical kinase elements include the G-loop (glycine-rich residues forming the active site roof), A-loop, or activation loop crucial for kinase activation and deactivation, C-helix responsible for the selectivity of the active site, and the hinge region that plays a vital role in inhibitor binding. The ATP-binding site categorized into five distinct regions: adenine region where the adenine moiety of ATP forms a hydrogen bond with the hinge, hydrophobic region-I corresponds to the entrance pocket, hydrophobic region-II also known as the selectivity pocket is determined by an extremely conserved DFG (aspartate-phenylalanine-glycine) motif, a hydrophilic region where the ATP sugar binds, and a solvent-exposed region for phosphate binding.

#### 2.4.4. Molecular Dynamic Simulation

Although X-ray crystallography and molecular docking are powerful techniques for biological macromolecules, they are unable to examine the protein’s flexibility and they provide a momentary binding mode. Therefore, MD simulation was used to investigate the ligand-protein interactions [36] and the conformational changes which occur in the structure of kinases with respect to motions. Based on the docking results, nine complexes were selected for further MD simulation to understand the binding mechanism of compound **10** and its stability against multiple kinases.

##### **c-Src** 

**Figure 8:**
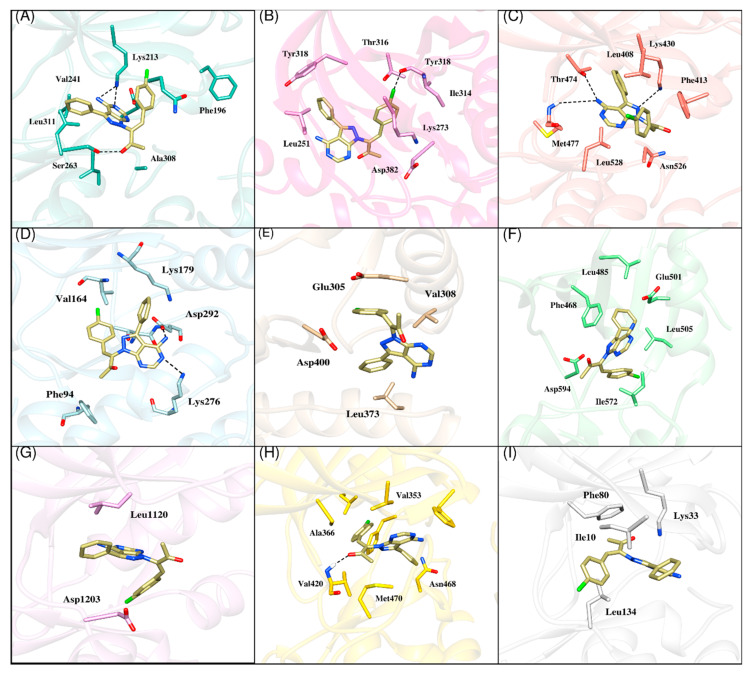
Binding mode of Z configuration of compound **10** (khaki) with the active site residues of (**A**) c-Src, (**B**) Lck, (**C**) Btk, (**D**) Akt, (**E**) Abl1, (**F**) Braf, (**G**) Alk, (**H**) PKC, and (**I**) Cdk2. Amino acid residues involved in crucial interactions are labeled.

The stereo view of the binding mode of Src (PDB ID: 2SRC) is depicted in Figure 8A. It was found that, initially, compound **10** was bound to the active site of Src kinase similar to a cognate ligand by making a hydrogen bond with the NH group of Met341 and CO group of Glu339, while these bonds were observed to undergo destabilization during the simulation. Comparatively, three new hydrogen bonds were formed, involving the side chain amino group of Lys295 (solvent accessible area) and the hydroxyl group of Ser345, which interact with the pyrazolopyrimidine scaffold located in the adenine region of Src kinase. The spatial position of the compound was found to be different from the observed dock pose because of major conformational changes during simulation.

The activation loop in the DFG-in conformation provides enough space to accommodate the ring of compound **10**, which may cause a collision with Phe405 of the DFG-out conformation. Moreover, distinct hydrophobic interactions were also observed with Leu273, and Val281 (hydrophobic region I) that maintain the binding mode of complexes. The MD results suggest that the non-flexible part of the compound might be the reason for its moderate activity as compared to the reference compound.

##### **Lck** 

The binding elements responsible for the modest activity of Lck kinase are depicted in Figure 8B. Consistent with the results from other reported studies, compound **10** is well accommodated in the ATP site of Lck by forming multiple hydrophobic, π-stacking, π-cation, and Van der Waals interactions with Leu251, Ala271, Lys273, Thr316, Lys318, and Asp382. Due to the different conformation of the DFG motif, compound **10** slightly moves toward the adenine region with its core pyrimidine ring in the adenine region by forming a hydrophilic interaction with Met319. In the meantime, Phe385 of the DFG motif is involved in a weak hydrophobic interaction due to the far distance. The chloro-benzene ring engages the gatekeeper residue Thr316 via hydrophobic interaction, while the non-substituted benzene ring occupies the extended hydrophobic pocket, forming π-stacking and Van der Waals interactions with Leu251, Ala271, and Tyr318. The complex was further stabilized by the formation of a halogen bond between the backbone of the carbonyl oxygen of Ile314 and the fluorine of the compound **10**. In summary, a marked similarity of interaction was observed with the reference compound except for the hydrophilic interaction responsible for the inhibitory activity of Lck kinase.

##### **Btk** 

The docked complex of BTK presented a number of hydrophilic and hydrophobic interactions with the active site residues Leu408, Val416, Lys430, Thr474, Glu475, Met477, Leu528, Asp539, and Phe540. The compound occupied the same position consistent with the reference compound. Molecular dynamics studies revealed that compound **10** binding to BTK, is stable and it was found to involve in hydrogen bond interactions with key residues of the glycine-rich P-loop like Leu408, Gly409, and Asp539. Transient hydrogen bond interactions were also detected with crucial active site residues Glu475 and Met477 of the hinge region, catalytic residue Lys430, and the DFG motif that is crucial for kinase activity, indicating that compound **10** probably has the appropriate interaction profile with BTK (Figure 8C).

##### **Akt** 

According to the binding mode of the Akt complex [37], compound **10** adopts a Y-shape conformation, where the presence of residues Phe469 and Phe472 that are essential for the regulatory control of Akt1, make the pocket hydrophobic. Initially, the pyrimidine ring occupies the adenosine portion of the binding site, forming hydrogen bonding to the hinge region of the kinase through Ala230 (Figure 8D). During the simulation, this hydrogen bond is replaced by hydrophobic interactions, while the phenyl substituent presents a parallel aromatic interaction with Phe438. Moreover, residues Lys179, Leu181, and Val164 also stabilize the compound by making hydrophobic contact. In general, theoretical studies indicate that compound **10** stabilizes Akt via hydrophobic interactions; however, after losing its interactions with the hinge region, the gatekeeper residue and the DFG motif lead to its low kinase inhibitory properties.

##### **Abl** 

The binding mode of the Abl1 complex (Figure 8E) showed that the compound is accommodated in the lipophilic channel slightly away from the hinge region. The bent conformation of the molecule does not allow its tight binding to the ATP site. This resultant compound is unable to mimic interaction with the adenine moiety of ATP. Only a few hydrophobic interactions were observed with Glu305, Val308, Leu473, and Asp400, but these interactions were also not found to be persistent throughout the md simulation. It is hypothesized that the absence of specific interactions of compound **10** with the residues of the hinge region and DFG motif could be the reason for its inactivity.

##### **Braf** 

According to the docking calculations, compound **10** is involved in a number of hydrophilic and hydrophobic interactions with Braf kinase. However, after a short 50 ns production run, the simulated binding mode provided a different picture to understand the selectivity trend. During the simulation, these non-bonded interactions were weakened due to loop flipping of the motif from the DGF-in to the DGF-out conformation. However, in order to accommodate the core pyrimidine ring, the benzene ring was required to tilt out of the plane due to restricted space. As a result of this movement, intermolecular interactions broke, which penalized the binding affinity of compound **10** for Braf (Figure 8F).

##### **Alk** 

The binding interactions of the Alk with compound **10** are depicted in Figure 8G. The complex obtained after simulation indicates that the compound partially aligned on the reference compound ceritinib, and it was unable to form any hydrophilic interaction with the active site residues, especially with the hinge region. A similar type of binding mode was observed in the case of PKC (Figure 8H) and Cdk2 (Figure 8I).

#### 2.4.5. Binding Free Energy Calculations

MM/PBSA is considered as the best choice for predicting the binding affinities of a protein-ligand complex with implicit solvent at a low computational cost. It has the potential to recognize the true binding structure of the complex from docking. In the present study, MM/PBSA was used to re-rank the nine complexes after a short production run of 50 ns. The calculated binding free energy of all complexes is presented in Table 4 [38]. The binding position and atomic interaction between protein kinases and ligand are the main factors that play a role in measuring the binding affinity. The predicted binding poses of compound **10** significantly overlaid with the reported inhibitors of c-Src, Lck, Btk, and Akt, but partially aligned in the case of Braf and Alk. According to Table 4, the binding free energy computed for c-Src, Lck, and Akt complexes was −20.63, −15.64, and −18.09, respectively, which were more stable than ALK, CDK2, and Abl1 kinases, agreeing with the selectivity profile observed experimentally. In order to comprehend the effect of the individual energy term in the binding process, total binding free energy was decomposed into the Eele, Evdw, EGB, and ESURF energy components. The values of these components indicated that van der Waals (∆Gvdw) energy contributed significantly to the total binding free energy. The superior contribution of Van der Waals interaction (Table 4) was in agreement with the fact that compound **10** mainly stabilizes via hydrophobic interactions with the kinases.

## 3. Experimental Protocols

### 3.1. Materials and Methods

Bromoacetone was purchased from ChemService Inc. (West Chester, PA, USA). Anhydrous dichloromethane, anhydrous pyridine, *N*,*N*-dimethylformamide (DMF), and other chemicals and reagents were purchased from Sigma-Aldrich Chemical Co. (Milwaukee, WI, USA). The chemical structures of final products were characterized by nuclear magnetic resonance spectra (^1^H NMR, ^13^C NMR) determined on a Bruker (Billerica, MA, USA) NMR spectrometer (400 MHz) or a Varian (Silicon Valley, CA, USA) NMR spectrometer (500 MHz). ^13^C NMR spectra were fully decoupled. Chemical shifts were reported in parts per millions (ppm) using deuterated solvent peak as the standard. The chemical structures of final products were confirmed by a high-resolution Biosystems QStar Elite (QStar Technologies, Denver, CO, USA) time-of-flight electrospray mass spectrometer. Details of procedures and spectroscopic data of the respective compounds are presented below. Final compounds were purified on a Phenomenex (Torrance, CA, USA) Prodigy 10 μm ODS reverse-phase column (2.1 cm × 25 cm) with a Hitachi (Tokyo, Japan) HPLC system using a gradient system of acetonitrile or methanol and water (CH_3_CN/CH_3_OH/H_2_O, 0−100%, pH 7.0, 60 min). The purity of final products (>99%) was confirmed by analytical HPLC. The analytical HPLC was performed on the Hitachi analytical HPLC system using a C18 Shimadzu (Kyoto, Japan) Premier 3 μm column (150 cm × 4.6 mm) using two different isocratic systems, and a flow rate of 1 mL/min with UV detection at 265 nm.

### 3.2. Synthesis

3-Phenyl-1*H*-pyrazolo[3,4-*d*]pyrimidin-4-amine (**1**): Compound **1** was synthesized according to a previously reported procedure [23].

1-(4-Amino-3-phenyl-1*H*-pyrazolo[3,4-*d*]pyrimidin-1-yl)propan-2-one (**2**): Compound **1** (156 mg, 0.73 mmol) and potassium carbonate (200 mg, 1.4 mmol) were taken in dry DMF (10 mL). Bromoacetone (100 mg, 0.72 mmol) was diluted with dry DMF (5 mL) and was added dropwise to the reaction mixture at room temperature. The reaction mixture was stirred for 4 h at room temperature. After completion of the reaction, the solvent was removed under reduced pressure, added water (50 mL) and extracted with dichloromethane. The organic layer was filtered through filter paper and dried over Na_2_SO_4_ (anhydrous). The crude product was further purified on silica gel flash chromatography using dichloromethane and methanol (0–20%) as eluents. 160 mg, 81% yield. ^1^H-NMR (CDCl_3_, 500 MHz, δ ppm): 8.3 (s, 1H), 7.7 (d, *J* = 11 Hz, 2H), 7.5–7.5 (m, 3H), 5.2 (s, 2H), 2.2 (s, 3H).^13^C NMR (CDCl_3_, 125 MHz, δ ppm): 201.3, 158.1, 156.2, 155.2, 145.4, 132.8, 129.4, 129.3, 128.4, 98.4, 56.1, 27.1. HR-MS (ESI-TOF) (*m*/*z*): calculated for C_14_H_13_N_5_O + H^+^: 268.1193; found, 268.0852 [M + H]^+^.

#### General Procedure for the Synthesis of Pyrazolopyrimidine Chalcones:

Compound **2** (26 mg, 0.1 mmol) was dissolved in ethanol (5 mL). Sodium hydroxide (5 mg, 20 µL of 250 mg/mL NaOH solution in water) was added to the compound **2** solution. The reaction mixture solution was stirred for 15 min. Then, a solution of *p*-methylbenzaldehyde (24 mg, 0.2 mmol) in ethanol (15 mL) was added to the reaction mixture. The reaction mixture was allowed to stir at room temperature for 5 h. On completion of the reaction, the reaction mixture was neutralized with dilute acetic acid in ethanol and purified over reverse phase HPLC using water and methanol as eluents to obtain pure product **3** (22 mg, 61% yield). A similar procedure was adopted for the synthesis of other pyrazolopyrimidine derivatives.

3-(4-Amino-3-phenyl-1*H*-pyrazolo[3,4-*d*]pyrimidin-1-yl)-4-*p*-tolylbut-3-en-2-one (**3**). (Yield: 61%). ^1^H-NMR (CDCl_3_, 500 MHz, *δ* ppm): 8.3 (s, 1H), 7.7 (s, 1H), 7.7 (d, *J* = 7 Hz, 2H), 7.6–7.5 (m, 3H), 7.0 (d, *J* = 8.2 Hz, 2H), 6.9 (d, *J* = 8.2 Hz, 2H), 2.3 (s, 3H), 2.9 (s, 3H). ^13^C NMR (CDCl_3_, 125 MHz, δ ppm): 194.3, 157.9, 156.9, 155.8, 146.8, 141.8, 140.5, 132.7, 131.8, 130.7, 129.6, 129.5, 129.4, 128.5, 26.1, 21.5. HR-MS (ESI-TOF) (*m*/*z*): calculated for C_22_H_19_N_5_O + H^+^: 370.1662; found, 370.1534 [M + H]^+^.

3-(4-Amino-3-phenyl-1*H*-pyrazolo[3,4-d]pyrimidin-1-yl)-4-*m*-tolylbut-3-en-2-one (**4**). (Yield: 64%). ^1^H-NMR (CDCl_3_, 500 MHz, δ ppm): 8.3 (s, 1H), 7.9 (s, 1H), 7.7 (d, *J* = 7 Hz, 2H), 7.6–7.5 (m, 3H), 7.1–7.0 (m, 2H), 6.8 (s, 1H), 6.7 (d, *J* = 10 Hz, 1H), 2.3 (s, 3H), 2.2 (s, 3H). ^13^C NMR (CDCl_3_, 125 MHz, δ ppm): 194.3, 157.8, 155.1, 154.9, 146.8, 140.6, 133.9, 131.7, 131.5, 130.2, 129.5, 129.4,128.6, 128.4, 26.2, 21.3. HR-MS (ESI-TOF) (*m*/*z*): calculated for C_22_H_19_N_5_O + H^+^: 370.1662; found, 370.1454 [M + H]^+^.

3-(4-Amino-3-phenyl-1*H*-pyrazolo[3,4-d]pyrimidin-1-yl)-4-*p*-biphenylbut-3-en-2-one (**5**). (Yield: 59%). ^1^H-NMR (CDCl_3_, 500 MHz, δ ppm): 8.0 (s, 1H), 7.9 (s, 1H), 7.7 (d, *J* = 8.2 Hz, 2H), 7.6–7.6 (m, 3H), 7.6–7.4 (m, 9H), 2.3 (s, 3H). ^13^C NMR (CDCl_3_, 125 MHz, δ ppm): 191.9, 147.2, 135.2, 131.1, 130.3, 129.9, 129.9, 129.8, 129.4, 129.0, 128.7, 128.5, 128.5, 127.7, 127.3, 127.3, 127.2, 127.1, 127.0, 126.1, 22.5. HR-MS (ESI-TOF) (*m*/*z*): calculated for C_27_H_21_N_5_O + H^+^: 432.1819; found, 432.1616 [M + H]^+^.

3-(4-Amino-3-phenyl-1*H*-pyrazolo[3,4-d]pyrimidin-1-yl)-4-(4-methoxyphenyl)but-3-en-2-one (**6**). (Yield: 63%). ^1^H-NMR (CDCl_3_, 500 MHz, δ ppm): 8.3 (s, 1H), 7.9 (s, 1H), 7.7 (d, *J* = 7 Hz, 2H), 7.6–7.5 (m, 3H), 6.9 (d, *J* = 8.9 Hz, 2H), 6.7 (d, *J* = 8.9 Hz, 2H), 3.7 (s, 3H), 2.3 (s, 3H). ^13^C NMR (CDCl_3_, 125 MHz, δ ppm): 194.2, 161.9, 158.0, 156.9, 155.7, 146.9, 140.2, 132.8, 132.7, 130.6, 129.5, 129.4, 128.5, 124.3, 114.4, 98.9, 55.3, 26.0. HR-MS (ESI-TOF) (*m*/*z*): calculated for C_22_H_19_N_5_O_2_ + H^+^: 386.1612; found, 386.1573 [M + H]^+^.

3-(4-Amino-3-phenyl-1*H*-pyrazolo[3,4-d]pyrimidin-1-yl)-4-(4-(dimethylamino)phenyl)but-3-en-2-one (**7**). (Yield: 66%). ^1^H-NMR (CDCl_3_, 500 MHz, δ ppm): 8.3 (s, 1H), 7.9 (s, 1H), 7.7 (d, *J* = 8 Hz, 2H), 7.6–7.5 (m, 3H), 6.8 (d, *J* = 9.1 Hz, 2H), 6.4 (d, *J* = 9.1 Hz, 2H), 2.9 (s, 6H), 2.3 (s, 3H). ^13^C NMR (CDCl_3_, 125 MHz, δ ppm): 194.4, 162.1, 158.2, 157.1, 155.9, 147.1, 140.4, 133.0, 132.9, 130.8, 129.7, 129.6, 128.7, 124.5, 114.6, 99.0, 41.4, 26.3. HR-MS (ESI-TOF) (*m*/*z*): calculated for C_23_H_22_N_6_O + H^+^: 399.1928; found, 399.1981 [M + H]^+^.

3-(4-Amino-3-phenyl-1*H*-pyrazolo[3,4-d]pyrimidin-1-yl)-pent-3-en-2-one (**8**). (Yield: 60%). ^1^H-NMR (CDCl_3_, 500 MHz, δ ppm): 8.37 (s, 1H), 7.8–7.7 (m, 2H), 7.6–7.5 (m, 3H), 7.4 (q, *J* = 7.1 Hz, 1H), 2.3 (s, 3H), 1.8 (d, *J* = 7 Hz, 3H). ^13^C NMR (CDCl_3_, 100 MHz, δ ppm): 157.9, 156.3, 154.8, 141.7, 132.6, 129.6, 129.5, 129.5, 128.5. 128.4, 98.3, 56.2, 26.1, 14.6. HR-MS (ESI-TOF) (*m*/*z*): calcd for C_16_H_15_N_5_O + H^+^: 294.1349; found, 294.099 [M + H]^+^.

3-(4-Amino-3-phenyl-1*H*-pyrazolo[3,4-d]pyrimidin-1-yl)-4-(3-nitrophenyl)but-3-en-2-one (**9**). (Yield: 44%). ^1^H-NMR (CDCl_3_, 500 MHz, δ ppm): 8.3 (s, 1H), 7.9 (s, 1H), 7.7 (d, *J* = 7 Hz, 2H), 7.5–7.5 (m, 3H), 6.9 (d, *J* = 8.9 Hz, 2H), 6.7 (d, *J* = 8.9 Hz, 1H), 2.3 (s, 3H). ^13^C NMR (CDCl_3_, 125 MHz, δ ppm): 194.2, 161.9, 158.0, 156.9, 155.7, 146.9, 140.2, 132.8, 132.7, 130.6, 129.5, 129.4, 128.5, 124.3, 114.4, 98.9, 55.3, 26.0. HR-MS (ESI-TOF) (*m*/*z*): calculated for C_21_H_16_N_6_O_3_ + H^+^: 401.1357; found, 401.0957 [M + H]^+^, 801.1652 [2M + H]^+^.

3-(4-Amino-3-phenyl-1*H*-pyrazolo[3,4-d]pyrimidin-1-yl)-4-(4-chlorophenyl)but-3-en-2-one (**10**). (Yield: 57%). ^1^H-NMR (CDCl_3_, 500 MHz, δ ppm): 8.1 (s, 1H), 7.9 (s, 1H), 7.6 (m, 2H), 7.5–7.5 (m, 3H), 7.2 (d, *J* = 8.6 Hz, 2H), 6.9 (d, *J* = 8.6 Hz, 2H), 2.4 (s, 3H). ^13^C NMR (CDCl_3_, 125 MHz, δ ppm): 192.4, 153.7, 153.5, 149.5, 147.2, 140.6, 137.9, 132.5, 131.5, 131.3, 130.9, 130.4, 130.1, 129.5, 128.1, 97.6, 55.3, 25.8. HR-MS (ESI-TOF) (*m*/*z*): calculated for C_21_H_16_ClN_5_O + H^+^: 390.1116; found, 390.0951 [M + 1]^+^.

3-(4-Amino-3-phenyl-1*H*-pyrazolo[3,4-d]pyrimidin-1-yl)-4-(2,4-dichlorophenyl)but-3-en-2-one (**11**). (Yield: 58%). ^1^H-NMR (CDCl_3_, 500 MHz, δ ppm): 8.3 (s, 1H), 7.9 (s, 1H), 7.7 (d, *J* = 7 Hz, 2H), 7.5–7.5 (m, 3H), 6.9 (s, 1H), 6.7 (d, *J* = 8.6 Hz, 2H), 2.6 (s, 3H). ^13^C NMR (CDCl_3_, 125 MHz, δ ppm): 192.2, 153.7, 136.9, 136.2, 130.9, 130.2, 130.1, 129.8, 129.0, 128.2, 128.1, 127.5, 127.5, 97.6, 66.4, 53.4, 25.9. HR-MS (ESI-TOF) (*m*/*z*): calculated for C_21_H_15_Cl_2_N_5_O + H^+^: 424.0726; found, 424.1220 [M + H]^+^.

3-(4-Amino-3-phenyl-1*H*-pyrazolo[3,4-d]pyrimidin-1-yl)-4-(2-chlorophenyl)but-3-en-2-one (**12**). (Yield: 57%). ^1^H-NMR (CDCl_3_, 500 MHz, δ ppm): 8.3 (s, 1H), 8.2 (s, 1H), 7.6–7.5 (m, 5H), 7.3–7.2 (m, 4H), 2.6 (s, 3H). ^13^C-NMR (CDCl_3_, 125 MHz, δ ppm): 153.6, 153.3, 149.1, 146.5, 138.2, 132.0, 130.8, 130.2, 130.1, 129.3, 128.9, 128.1, 126.9, 52.5, 25.9. HR-MS (ESI-TOF) (*m*/*z*): calcd for C_21_H_16_ClN_5_O + H^+^: 390.1116; found, 390.1427 [M + H]^+^.

3-(4-Amino-3-phenyl-1*H*-pyrazolo[3,4-d]pyrimidin-1-yl)-4-(2-fluorophenyl)but-3-en-2-one (**13**). (Yield: 62%). ^1^H-NMR (CDCl_3_, 500 MHz, δ ppm): 8.3 (s, 1H), 7.9 (s, 1H), 7.7 (d, *J* = 7 Hz, 2H), 7.6–7.5 (m, 3H), 7.2–6.9 (m, 4H), 2.3 (s, 3H). ^13^C NMR (CDCl_3_, 125 MHz, δ ppm): 193.8, 158.1, 155.6, 147.4, 132.8, 132.7, 130.3, 131.7, 129.7, 129.5, 129.5, 129.3, 129.2, 128.4, 128.4, 128.4, 98.7, 26.2. HR-MS (ESI-TOF) (*m*/*z*): calculated for C_21_H_16_FN_5_O + H^+^: 374.1412; found, 374.1526 [M + H]^+^.

3-(4-Amino-3-phenyl-1*H*-pyrazolo[3,4-d]pyrimidin-1-yl)-4-(4-fluorophenyl)but-3-en-2-one (**14**). (Yield: 64%). ^1^H-NMR (CDCl_3_, 500 MHz, δ ppm): 8.1 (s, 1H), 7.8 (s, 1H), 7.5–7.4 (m, 5H), 6.9 (dd, *J* = 14 Hz, *J* = 5.6 Hz, 2H), 6.8 (t, *J* = 7.6 Hz, 2H), 2.4 (s, 3H). ^13^C NMR (CDCl_3_, 125 MHz, δ ppm): 192.5, 153.7, 153.5, 147.2, 140.7, 132.5, 132.4, 130.9, 130.1, 130.0, 128.1, 128.1, 116.7, 116.5, 25.7. HR-MS (ESI-TOF) (*m*/*z*): calculated for C_21_H_16_FN_5_O + H^+^: 374.1412; found, 374.1679 [M + H]^+^.

3-(4-Amino-3-phenyl-1*H*-pyrazolo[3,4-d]pyrimidin-1-yl)-4-phenyl but-3-en-2-one (**15**). (Yield: 59%). ^1^H-NMR (CDCl_3_, 500 MHz, δ ppm): 8.2 (s, 1H), 8.0 (s, 1H), 7.7–7.6 (m, 5H), 7.4–6.9 (m, 5H), 2.5 (s, 3H). ^13^C-NMR (CDCl_3_, 125 MHz, δ ppm): 192.5, 153.7, 153.5, 147.2, 140.7, 132.5, 132.4, 130.9, 130.1, 130.0, 128.1, 128.1, 116.7, 116.5, 25.7. HR-MS (ESI-TOF) (*m*/*z*): calculated for C_21_H_17_N_5_O + H^+^: 356.1506; found, 356.1703 [M + H]^+^.

### 3.3. Kinase Enzyme Assay

#### 3.3.1. c-Src Kinase Activity Assay

The effect of compounds on the activity of *c*-Src kinase was assessed using a Transcreener^®^ ADP [2,8] fluorescence intensity (FI) Assay, from Bell Brook Labs, Madison, WI, USA, (catalog no. 3013-1K) according to the manufacturer’s protocol. A 384-well low-volume black non-binding surface round-bottom microplate was purchased from Corning Inc. (Corning, NY, USA) (#3676). In summary, the kinase reaction was started in the 384-well low volume black microplate with the incubation of 2.5 μL of the reaction cocktail (0.7 nM of His_6_-Src kinase domain in kinase buffer) with 2.5 μL of prediluted compounds (dissolved in 10% DMSO, 4× target concentration) for 10 min at room temperature using microplate shaker. The reaction cocktail was made using the kinase buffer HEPES (200 mM, pH 7.5), MgCl_2_ (16 mM), EGTA (8 mM), DMSO (4%), Brij-35 (0.04%), and 2-mercaptoethanol (43 mM). The kinase reaction was started by adding 5 μL of ATP/substrate (40 μM/600 μM) cocktail and incubated for 30 min at room temperature on a microplate shaker. Src optimal peptide (AEEEIYGEFEAKKKK) was used as the substrate for the kinase reaction. The kinase reaction was stopped by adding 10 μL of the 1× ADP Detection Mixture to the enzyme reaction mixture and mixed using a plate shaker. The mixture was incubated at room temperature for 1 h, and the fluorescence intensity was measured. The 1× ADP Detection Mixture was prepared by adding ADP^2^ Antibody-IRDyeR QC-1 (10 μg/mL) and ADP Alexa594 Tracer (8 nM) to Stop and Detect Buffer B(1×). The fluorescence Intensity measurements were performed using a fluorescence intensity optical module using the excitation of 580 nm and emission of 630 nm with bandwidths of 10 nm by Optima, BMG Labtech microplate reader (BMG Labtech, Ortenberg, Germany). The IC_50_ values of the compounds were calculated using ORIGIN 6.0 (Origin Lab, Microcal Software, Inc. Northhampton, MA, USA) software. IC_50_ is the concentration of the compound that inhibited enzyme activity by 50%. All the experiments were carried out in triplicate.

#### 3.3.2. Evaluation of Compound **10** Against a Panel of Kinases (Abl1, Akt1, Alk, Braf, Btk, Cdk2, cSrc, Lck, and PKCa)

Compound **10** was evaluated for inhibitory activity against Abl1, Akt1, Alk, Braf, Btk, Cdk2, cSrc, Lck, and PKCa, in duplicate by Reaction Biology Corporation. Compounds were tested in a five-dose IC_50_ mode with four-fold serial dilution starting at 20 µM. Control compounds Staurosporine and GW5074 were tested in a 10-dose IC_50_ starting at 20 µM with a four-fold or three-fold serial dilution, respectively. All kinase reactions were performed at 200 µM ATP. The compounds were pre-incubated with the enzyme and substrate mixture about 20 min, and then ATP was added to start the reaction. The reaction was 2 h at room temperature. Curve fits were performed when the activities at the highest concentration of compounds were less than 65%. The detailed experimental procedure is provided here. An IC_50_ value of less than 0.04 nM or higher than 1 µM is estimated based on the best curve fitting available. The substrates for kinases are shown in Table 5.

Base Reaction buffer was prepared from 20 mM Hepes (pH 7.5), 10 mM MgCl_2_, 1 mM EGTA, 0.02% Brij35, 0.02 mg/mL BSA, 0.1 mM Na_3_VO_4_, 2 mM DTT, and 1% DMSO. Required cofactors were added individually to each kinase reaction. Testing compounds were dissolved in 100% DMSO to a specific concentration. The serial dilution was conducted by Integra Viaflo Assist in DMSO. The substrate was prepared in a freshly prepared reaction buffer. The required cofactors for each kinase were added to the substrate solution. The kinase was added into the substrate solution, and the solution was gently mixed. The compounds in 100% DMSO were added into the kinase reaction mixture by Acoustic technology (Echo550; nanoliter range) and incubated for 20 min at room temperature. P-ATP (Specific activity 10 µCi/µl) was added into the reaction mixture to initiate the reaction. The mixture was incubated for 2 h at room temperature. The radioactivity was detected using the filter-binding method. The kinase activity data were expressed as the percentage remaining kinase activity in test samples compared to vehicle (dimethyl sulfoxide) reactions. IC_50_ values, and curve fits were obtained using GraphPad Prism software (version 7, Informer Technologies, Inc., Los Angeles, CA, USA).

### 3.4. Cell Culture and Cell Proliferation Assay

#### 3.4.1. Cell Culture

Human leukemia cell line CCRF-CEM (ATCC no. CCL-119), human ovarian adenocarcinoma cell line SK-OV-3 (ATCC no. HTB-77), human breast carcinoma cell line MDA-MB-231 (ATCC no.HTB-26), and human colon adenocarcinoma cell line HT-29 (ATCC no. HTB-38) were obtained from American Type Culture Collection. Cells were grown on 75 cm^2^ cell culture flasks with RPMI-16 medium (for leukemia cells) and EMEM (Eagle’s minimum essential medium) (for SK-OV-3, HT-29, MDA-MB-231 cells), supplemented with 10% fetal bovine serum (FBS), and 1% penicillin-streptomycin solution (10,000 units of penicillin and 10 mg of streptomycin in 0.9% NaCl) in a humidified atmosphere of 5% CO_2_, 95% air at 37 °C.

#### 3.4.2. Cell Proliferation Assay

The cell proliferation assay was carried out using a CellTiter 96 aqueous one-solution cell proliferation assay kit (Promega, Madison, WI, USA). Briefly, upon reaching about 75–80% confluence, 5000 cells/well were plated in 96-well microplate in 100 μL of the medium. After seeding for 24 h, the cells were treated with 50 µM compound in triplicate. Doxorubicin (10 µM) was used as a positive control. At the end of the sample exposure period (72 h), 20 µL of CellTiter 96 aqueous solution was added. The plate was returned to the incubator for 1 h in a humidified atmosphere at 37 °C. The absorbance of the formazan product was measured at 490 nm using a microplate reader. The blank control was recorded by measuring the absorbance at 490 nm with wells containing medium mixed with CellTiter 96 aqueous solution but no cells. The percentage of cell survival was calculated as the OD value of cells treated with test compound − OD value of culture medium/(OD value of control cells − OD value of culture medium) × 100%. The same strategy was used for generating a dose-response graph for compound **10**.

### 3.5. Molecular Modeling

#### 3.5.1. Target Fishing

Potential targets were identified against the phenylpyrazolopyrimidine scaffold using a combination of information integration, chemometric methods, and data mining approaches. Initially, targets involved in breast, ovarian, and colorectal cancer pathways were extracted from the database of KEGG. In the next step, protein network association servers STRING, STITCH, and SEA were used to characterize the protein-ligand interactions. Similarly, the PhPP scaffold was also searched using different search engines to understand protein-ligand interactions. Based on the above-mentioned information, we selected fourteen different targets, including Btk, Itk, c-Src, EGFR [39], Braf, Fyn, Lyn, Hck, Cdk2/4, Alk, Akt1 Lck, PKC, and Abl1 kinase. To understand the binding mechanism of active compound **10** against all selected targets, molecular docking and MD simulations were performed.

#### 3.5.2. Preparation of Complexes

Crystal structures of the Anaplastic lymphoma kinase [PDB ID: 4MKC], ABL1 kinase [PDB ID: 6HD4], Protein kinase B also known as AKT [PDB ID: 4GV1], bruton tyrosine kinase [PDB ID: 6DIS], BRAF [PDB ID: 6B8U], c-Src kinase [PDB ID: 2SRC], CDK2 [PDB ID: 4KD1], LCK [PDB ID: 2OFU], ITK [PDB ID: 4HUC], EGFR [PDB ID: 4HJO], FYN [PDB ID: 2DQ7], LYN [PDB ID: 2ZV9], HCK [PDB ID: 1QCF], and PKCa [PDB ID: 3IW4] were retrieved from RCSB Protein Databank. The missing part of the starting structures was constructed using MOE software (Molecular Operating Environment, v2016.08, 2016). Compound partial charges were derived with the MMFF94x force field. All stabilizing agents, water molecules, and ions present in the crystal structures were removed. The protonate 3D module in MOE was used for assigning the protonation state to every titratable residue at physiological conditions within the complexes. MOE docking suite was employed for docking studies. The standard default settings were used with the London dG scoring function as a fitness function in all calculations.

#### 3.5.3. MD Simulations

After analysis of docking results, nine complexes were subjected to all-atom MD simulation using the graphics processing unit (GPU) version of the PMEMD.cuda engine provided with Amber18 [40]. The gaff [41] and ff14SB force field [42] were implemented to ligand and protein in all simulations, respectively. Each complex was neutralized by adding counter ions, and the systems were solvated by a cubic box of the TIP3P water model with a 10 Å box size from the solute. Systems were relaxed by adjusting hydrogen position, while 1000 cycles of steepest descent, and 5000 cycles of the conjugate gradient with restraints of 100 kcal·mol^−1^ were applied on heavy atoms. Finally, another minimization of 5000 steps was performed to relax the system without any restraint. It was followed by equilibration of each system by gradual heating from 0 K to 300 K in the NVT ensemble. Systems were further equilibrated under the NPT ensemble (Pressure = 1 atm). The production run of 50 ns was performed for each system using the isothermal-isobaric (NPT) ensemble at constant temperature and pressure with periodic boundary conditions. The Particle Mesh Ewald Method (PMEM) [43] was used to calculate long-range electrostatic interactions with cut off value of 8 Å for the non-bonded (electrostatics and Van der Waals) interactions. An integration time step of 2 fs was set to integrate Newton’s equations of motion. The SHAKE algorithm [44] was employed to restrain the bonds involving hydrogen atoms. The trajectories were analyzed using CPPTRAJ module incorporated in AMBER suite. All visualization was performed using Chimera and VMD.

#### 3.5.4. MM/PBSA Calculations

The binding free energy calculation for each complex was performed using the MM(GB/PB)SA method based on 1000 frames extracted from the 50 ns trajectory. The equations associated with binding free energy calculation were as follows:

ΔG = G_complex_ − (G_receptor_ + G_compound_) (1)

G = H − TS(2)

H = E_vdw_ + E_ele_ + E_int_ + G_gb_ + G_surf_ (3)

The total binding free energy is ΔG, whereas the free energies for the complex, receptor, and compound are represented as G_complex_, G_receptor,_ and G_compound_, respectively. The terms H and TΔS were used for the contribution of enthalpy and entropy. The binding free energy further decomposed into electrostatic (E_ele_), Van der Waals interaction (E_vdw_), internal energy (E_int_), and polar (G_gb_) and non-polar desolvation energy (G_surf_). The value of the dielectric constant was set to 1, and the external was set to 80 for solute protein (εin). The Generalized Born (GB) model established by Onufriev (GBOBC1) with modified Bondi radii (mbondi2) and igb = 2 implemented in AMBER18 was used to calculate the electrostatic contribution to the solvation free energy. The non-polar surface area was calculated using the LCPO model. The offset (β) and surface tension (γ) values were set to 0.92 kcal/mol and 0.00542 kcal/(mol·Å2), respectively.

## 4. Conclusions

A number of novel *N*1-(α,β-alkene) substituted phenylpyrazolopyrimidine (PhPP) derivatives with acetyl and functionalized phenyl groups at α- and β-positions were synthesized, and they showed modest inhibitory activity (IC_50_ = 21.7–192.1 µM) against c-Src kinases. A number of para-chlorophenyl substituted compounds, **10** and **11,** consistently inhibited the cell proliferation 72–96 h in all cell lines and were more effective against HT-29 cells. Compound **10** was also found to be modestly active against c-Src, Lck, and Btk. The target-based study of these compounds was performed by text mining, similarity search, and ligand-protein interaction pattern to identify potential targets. Further MD simulation and binding free energy calculations were carried out to discriminate the selectivity of the investigated compound with a panel of different kinases and provide more in-depth structural insight into the interaction pattern involved in the inhibition process. The structural and energetic features highlighted in this study can provide a theoretical basis for further research on selective protein kinase inhibitors.

## Figures and Tables

**Table 1:** In vitro Src kinase inhibition assay for the selected compounds.

Compound No.	IC_50_ (µM) ^a^
**3**	32.9
**4**	24.7
**5**	192.1
**6**	21.7
**7**	36.9
Staurosporine	0.6
PP2	0.5

^a^ All experiments were repeated in triplicate. All the standard deviations were in the range of 6–13%.

**Table 2:** The activity of compound **10** against a panel of protein kinases.

Kinase	Compound 10 IC_50_ (µM)	Control Compound IC_50_ (µM)
Abl1	NA^a^	0.85 ^b^
Akt1	>150	0.012 ^b^
Alk	NA	0.01 ^b^
Braf	NA	0.018 ^c^
Btk	95.4	0.067 ^b^
Cdk2/Cyclin A1	NA	0.0039 ^b^
c-Src	64.1	0.0085 ^b^
Lck	110	0.011 ^b^
PKCa	NA	0.0016 ^b^

^a^ Not active (NA) indicates no inhibition or compound activity that could not be fit to an IC_50_ curve; ^b^ Staurosporine; ^c^ GW5074.

**Table 3:** Predicted binding affinity (docking scores in kcal/mol) and root-mean-square deviation (RMSD) of control inhibitors against related proteins.

Target Proteins	Docking Score of Comp. 10	RMSD (Å)
c-Src	−8.3240	1.5
Btk	−7.6201	0.75
Itk	−8.1748	1.4
EGFR	−7.7890	1.56
Fyn	−7.5002	1.32
Lyn	−5.9252	1.23
Hck	−5.1107	0.9
Her2	−8.7522	0.54
Lck	−7.7834	0.45
PKC	−6.4224	0.82
Braf	−6.8762	1.52
Abl1	−6.7421	0.85
Akt1	−7.8654	0.74
Cdk2	−5.2409	0.95

**Table 4:** MM/PBSA derived binding free energies of complexes computed from the MD simulations.

	c-Src	PKC	Akt	Alk	Btk	CDK2	Lck	Abl1	Braf
VDWAALS	−40.343	−46.197	−35.635	−35.533	−46.946	−50.067	−34.248	−40.962	−31.802
EEL	−13.296	−19.577	−15.181	−3.935	−13.128	−29.554	−15.558	−18.300	−10.603
EPB/EGB	32.183	39.385	33.442	23.836	36.678	54.064	32.371	39.952	26.077
ESURF/ECAVITY/ENPOLAR	−5.024	−5.873	−3.961	−4.54	−5.820	−6.421	−4.623	−5.313	−4.283
DELTA G binding (PB)	−20.633	−21.653	−15.645	−5.210	−15.683	−6.893	−18.093	−9.629	−20.433

**Table 5:** Substrates used for kinases.

Kinases	Kinase (Invitrogen) Cat#	Kinase Conc. in Reaction (nM)	Substrate	Substrate Conc. in Reaction
Abl1	PR4348B	0.25	ABLtide	20 µM
c-Src	P3044	0.6	pEY	0.2 mg/mL
Akt1	PR3878D	8	Crosstide	20 µM
Alk	PV3867	1.5	pEY	0.2 mg/mL
Cdk2/Cyclin A1	C29-10BG	15	Histone H1	20 µM
PKCa	PR1455C	0.5	Histone H1 + Lipid Activator	20 µM
Braf	PR6995A	30	MEK1 (K97R)	5 µM
Lck	P3043	8	pEY + Manganese	0.2 mg/mL
Btk	PV3363	8	pEY	0.2 mg/mL
